# Menthol and related compounds in waterpipe products

**DOI:** 10.18332/tpc/177170

**Published:** 2024-02-09

**Authors:** Ingrid Μ.Ε. Bakker-'t Hart, Frank Bakker, Jeroen L.Α. Pennings, Reinskje Talhout

**Affiliations:** 1 Centre for Health Protection, National Institute for Public Health and the Environment, Bilthoven, Utrecht, The Netherlands

**Keywords:** waterpipe, shisha, flavor, TRPM8, menthol, cooling

## Abstract

**INTRODUCTION:**

The addition of cooling substances, such as menthol, might be attractive for youth to start smoking waterpipe by reducing the harshness of the smoke, thereby facilitating inhalation. These compounds simultaneously increase the addictiveness of tobacco and related products by stimulating nicotine uptake. Some menthol-like compounds also increase attractiveness by imparting a menthol/mint flavor. We provide an overview of the frequency and quantities of use of menthol-like substances in waterpipe tobacco, herbal molasses and steam stones.

**METHODS:**

The primary data source of this study was the European Common Entry Gate (EU-CEG). Product names and ingredients were obtained for 282 waterpipe tobacco products notified to The Netherlands in 2020. Subsequently, gas-chromatography-mass spectrometry (GC-MS) analysis was used to quantify seven menthol-like substances and nicotine in waterpipe tobacco (n=5), herbal molasses (n=1) and steam stones (n=12).

**RESULTS:**

Of the 282 EU-CEG-notified products, 39% have a menthol/mint declared flavor. GC-MS showed that 15 of the 18 investigated waterpipe products contained one or more menthol-like ingredients. GC-MS analysis showed that products termed ‘freeze’, ‘ice’ or ‘mint’ contained higher median menthol concentrations than products without these terms.

**CONCLUSIONS:**

Nearly all investigated waterpipe products contained menthol-like compounds, irrespective of their flavor. Such compounds are known to provide flavoring or cooling effects, and some are known to be carcinogenic. Our results can support the regulation of these substances in waterpipe products. Regulators should screen all waterpipe products, not only those with menthol or a similar indicator in product names.

## INTRODUCTION

It is well documented that tobacco and nicotine products are harmful to health^1,2^. Therefore, regulations, such as the EU Tobacco Products Directive (TPD), were developed to protect human health, especially young people^3^. The TPD applies to the EU Member States and contains rules on packaging, contents, and emissions of tobacco and related products, such as herbal products and e-cigarettes. Due to market developments in nicotine and tobacco products and to meet the international aims of reducing tobacco use and smoking, the TPD will be revised.

The TPD Articles 7.6 and 20.3 ban all compounds that facilitate inhalation or nicotine uptake in tobacco products and e-cigarettes^3^. This is because such compounds reduce the harshness of the smoke or aerosol, and simultaneously increase the addictiveness. Recently, lists of compounds that facilitate inhalation and nicotine uptake were drawn up by various EU countries to aid the enforcement of these additives^4^.

Many compounds facilitate inhalation and nicotine uptake via different mechanisms^5^. This article focuses on cooling substances, such as menthol^6,7^. Other structurally similar menthol-like compounds, whose cooling properties are unknown but impart a menthol/mint flavor, are also included^7,8^.

The monoterpene menthol is a significant ingredient in mint extracts and contributes to a fresh taste^6^. Although primarily known to be added as a flavoring agent, menthol has also been found in various concentrations in tobacco products not marketed as menthol products^9^. In addition to providing flavor, menthol causes a cooling effect by binding to transient receptor potential melastatin 8 (TRPM8)^10^. Due to the cooling properties of menthol, irritation and harshness of smoke are reduced in tobacco products, thereby facilitating inhalation^11^. Furthermore, menthol is known to stimulate nicotine uptake and cause increased nicotine dependence^11^. Facilitation of inhalation and nicotine dependence by added menthol is especially concerning for new/experimental smokers such as youth. It has been reported that cooling effects can be achieved by concentrations far below those that impart any characterizing flavor^9,11-13^.

Other compounds, often structurally similar to menthol and hereafter named ‘menthol-like’ compounds, can contribute to a minty taste and provide similar cooling effects. Examples of menthol-like compounds are pulegone, R-carvone, menthone and *n*-ethyl-*p*-menthane-3-carboxamide (also known as WS-3)^6-8^.

For cigarettes and e-cigarettes, some research has been carried out on the presence of menthol and menthol-like compounds, using data retrieved from analytical-chemical studies or other sources, such as manufacturers’ data^14,15^.

It is also important to investigate the use of menthol and other cooling agents and flavorings in waterpipe products since these are often preferred over non-flavored tobacco and smoked by youth^16-18^. Waterpipe products frequently contain menthol/mint flavors, sometimes as the sole flavor but mainly as a secondary flavor (such as ‘blueberry mint’)^19^. So far, little research has been done on menthol-like ingredients in waterpipe products^19-21^.

The European Common Entry Gate (EU-CEG) system and additional gas-chromatography-mass spectrometry (GC-MS) analysis have previously been shown to provide an excellent basis for obtaining information on flavorings in general and are likely to provide more information on these menthol-like ingredients^19^. Manufacturers need to use the EU-CEG system to notify the country’s national regulator where they want to market all products that the TPD covers.

Only scarce data are available on the analytical-chemical detection of menthol-like compounds, other than menthol, in waterpipe tobacco and related products^13,20,21^. In particular, identifying these menthol-like substances often lacks confirmation by analytical standards and menthol-like substances besides menthol are rarely quantified^20,21^. Apart from testing for menthol in our previous study, no studies have ever been carried out on the detection of other menthol-like compounds in steam stones^19^.

Waterpipe is generally smoked by heating flavored or non-flavored tobacco^16^. Different alternative waterpipe products exist, such as herbal molasses or steam stones. Herbal molasses usually contains a plant- or fruit-based matrix other than tobacco, are mixed with flavors and are nicotine-free^22^. Steam stones are small porous mineral rocks dispersed in flavored glycerol-based liquid and are generally nicotine-free but are also offered with added nicotine^23,24^.

In this study, we provide an overview of menthol-like compounds used as additives in waterpipe tobacco and their average concentrations, based on analysis of EU-CEG data. GC-MS analysis was performed in this study for waterpipe tobacco and non-registered products, such as steam stones and herbal molasses. This provided a good method to quantify these menthol- and related ingredients present in these products. Results from this study will aid policy making and enforcement on the regulation of menthol and related ingredients in all types of waterpipe products.

## METHODS

Assignment of flavor categories to waterpipe tobacco products registered in the Dutch EU-CEG, June 2020

In 2020, 282 waterpipe tobacco products were notified to the Dutch EU-CEG database. Waterpipe products were assigned to leading- and sub-flavor categories based on their product descriptions and brand names in a similar way as earlier studies^19^.

### Identification of menthol-like ingredients in waterpipe tobacco products registered in the Dutch EU-CEG, June 2020

The 282 registered waterpipe tobacco products contained 421 unique ingredients based on CAS numbers (unique identification numbers for chemical substances assigned by the Chemical Abstracts Service). Among these ingredients, 22 menthol-like substances were identified based on their names (expert opinion), cooling properties, and/or flavoring descriptions from the Leffingwell (Leffingwell & Associates, Flavor-Base 9 - Tobacco Version for Windows XP/Vista/7&8 2013) and thegoodscentscompany.com databases. For these substances, CAS numbers, names, median registered concentrations, frequency of use in registered waterpipe tobacco, and flavor descriptions are presented in the Supplementary file Table S1, where the median concentrations are based on the values registered in EU-CEG by manufacturers. Data processing and analysis of EU-CEG data were performed by the statistical software program R (version 3.6.0) and Excel as described before^19^.

### Inclusion and exclusion criteria of menthol-like substances for GC-MS analysis

The following natural extracts were excluded for chemical analysis: cornmint oil, peppermint oil, spearmint oil, mint oil and spearmint absolute, since these are mixtures of chemicals. No distinction was made between stereo-isomers, so one standard (either an isomer or mixture of isomers) was chosen to represent all stereoisomers of a compound, resulting in the exclusion of DL-menthol and DL-isomenthone. D-carvone was used instead of L-carvone, which are standards with different flavor characteristics, therefore results are reported as ‘carvone’. No analytical standards were available for two substances and therefore excluded from chemical analysis: l-menthol ethylene glycol carbonate, and menthol 1- and 2-propylene glycol carbonate. Nicotine was included for GC-MS analysis, resulting in a total of 14 standards for GC-MS identification and quantification (menthol-like compounds are highlighted in grey in Supplementary file Table S1).

### Selection of waterpipe products for GC-MS analysis

Products with and without ‘mint’, ‘ice’, or ‘freeze’ in their product names were selected among waterpipe tobacco, herbal molasses and steam stones, and obtained from commercial vendors in The Netherlands (Table 1). This resulted in 5 waterpipe tobacco samples with different flavors (2 brands), 12 steam stones in 7 different flavors (3 brands, of which one brand contained two samples with added nicotine according to the vendor website and sticker on packaging material) and one herbal molasses. Of the 18 products, 9 contained ‘mint’, ‘ice’, or ‘freeze’ in their product names or product descriptions.

**Table 1:** Gas chromatography-mass spectrometry analysis of 7 identified menthol-like flavorings and nicotine, in 18 waterpipe products. Concentrations in mg/g are given as an average of n=2 with relative standard deviations (%). Limit of quantification, LOQ=0.07 mg/g. Main, secondary and tertiary flavors are given for each waterpipe product, and descriptions related to cooling compounds or menthol/mint flavors are presented as Freeze, Ice or Mint

*Product code*	*Main flavor*	*Secondary and tertiary flavors*	*Menthone*	*Carvone*	*Menthol*	*Menthyl acetate*	*Piperitone*	*Pulegone*	*Isopulegol*	*Nicotine*
**Tobacco**										
T9	Blueberry	Passion fruit *Ice*		2.34 (7)	7.01 (3)					1.54 (4)
T10	Mango	Pineapple *Mint*		1.68 (6)	4.18 (4)					1.26 (0)
T11	Strawberry									1.09 (1)
T12	Grape	*Mint*								1.00 (3)
T13	Blueberry				0.73 (1)	<0.07				0.91 (3)
**Steam stones**										
S5	Lemon	*Ice*	0.14 (8)	0.08 (4)	0.52 (0)					
S6	Cherry				0.22 (32)					
S7	Pineapple				0.32 (1)					
S8	Lemon	Cake		<0.07						
S9	*Ice*				0.68 (9)					
S10	Grape	*Mint*	<0.07	<0.07	1.39 (7)	<0.07				
S11	Grape		<0.07	<0.07	1.48 (17)	<0.07	<0.07	<0.07		
S12	Lemon									
S13	Lemon	*Mint*		<0.07	0.64 (18)					
S14	Peach	*Freeze*	<0.07	<0.07	0.65 (2)	0.17 (1)	0.10 (2)		<0.07	
S15^a^	Peach	*Freeze*	<0.07	<0.07	0.69 (6)	0.17 (7)	0.10 (9)		<0.07	
S16^a^	Blueberry				0.32 (4)					
**Herbal molasses**										
M8	Cherry			<0.07	0.44 (3)					

a Steam stones with added nicotine according to vendor website and sticker on packaging material.

### Chemicals for GC-MS analysis

Methanol ULC/MS was obtained from Biosolve (Valkenswaard, The Netherlands). Standards of the menthol-like substances were of analytical or food-grade purity (≥95%). Flavorings were purchased from Sigma-Aldrich (Zwijndrecht, the Netherlands), and ethyl acetate was purchased from Alfa Aesar (Kandel, Germany). Nicotine (purity=96%) and the internal standard benzene-d6 (purity=100%) were purchased from Acros Organics (Geel, Belgium) and Sigma-Aldrich, respectively.

### Standard solutions

For qualification of the flavorings and nicotine, the standards were individually dissolved in methanol (ca. 5 mg/mL), with the exception of piperitone (ca. 1 mg/mL). A solution of the internal standard benzene-d6 was prepared in ethanol (100 μg/mL). All menthol-like substance standards and nicotine were dissolved as a mixture in the internal standard solution in 5 different concentrations.

### Product extraction

Products from this study were extracted in a similar way as previously described, with the main difference that similarly methanol was used as extraction solvent instead of ethanol^19^. Briefly: 3 g (± 10%) of each waterpipe product were weighed in a 50 mL tube (in duplicate); 20 mL of methanol containing 100 μg/mL of the internal standard was added and the mixture was shaken for 60 min at room temperature. An aliquot (500 μL) was taken out and centrifuged in an Eppendorf tube and passed through a 0.45 μm syringe filter. The filtrate was further diluted (10×) with methanol before GC-MS analysis.

### GC-MS conditions and data analysis

An Agilent 7890B GC system coupled with an Agilent 240 ion trap mass spectrometer was used, equipped with a 7693 auto-sampler and a G4513A injector. Data processing and analysis were performed by the MS workstation (version 7.0.2, Agilent technologies). GC-MS runs and data analysis were performed in the same manner as described before^19,25^. The 13 selected menthol-like substances and nicotine were quantified (by quantifier ion of analytical standard) in waterpipe samples where the respective flavoring was positively identified (by qualifier ion of analytical standard). Concentrations of the menthol-like substances are averages of duplicate extractions. Limits of detection (LOD) were calculated based on the calibration curve as S/N 3/1; the limit of quantification (LOQ) was set as the lowest point of the calibration curve (10 μg/mL).

## RESULTS

### Data analysis on 2020 EU-CEG registered waterpipe tobacco products in The Netherlands

**Figure 1:**
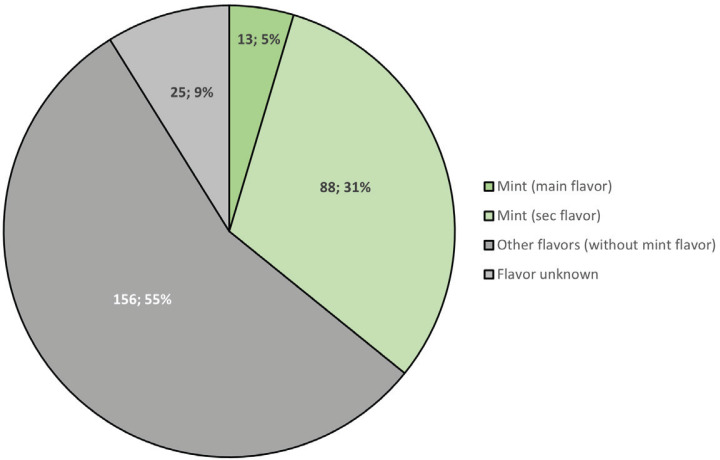
Flavor categorization of waterpipe tobacco products (N=282; 100%) registered in the Dutch European Common Entry Gate (EU-CEG) database, June 2020. No information was available for 25 waterpipe tobacco products (9%) to assign a flavor. Menthol/mint flavor could be assigned to 5% of the registered products as the main flavor. The main flavor of a waterpipe product was the first flavor mentioned in EU-CEG or on the vendor website. Menthol/mint flavor could be assigned to 31% of the registered products as secondary or tertiary flavor. The remaining products (55%) did not contain menthol/mint flavors in their primary or secondary flavor descriptions

In 2020, 282 waterpipe tobacco products were registered in the Dutch EU-CEG system. For 25 of the registered products, too limited information was registered to be able to assign to a flavor category (Figure 1). Of the remaining products (257), 13 contained menthol/mint as the first mentioned flavor in the product name or description, and 88 had a menthol/mint flavor as a secondary flavor (not mentioned as the first flavor in the description). In total, 39% of the categorized registered products contained menthol/mint in their product names or flavor descriptions.

### Added ingredients as registered in EU-CEG

Supplementary file Table S1 provides an overview of the menthol-like substances, their prevalence in waterpipe tobacco products and the median concentrations in which they are added to waterpipe tobacco.

DL-Menthol was noted in the EU-CEG database in the highest median amount (13.5 mg/g), followed by menthol-containing oils (4.5–1.5 mg/g) and L-carvone (2.33 mg/g). In comparison, isopulegol was noted to be the lowest amount (0.004 mg/g).

### Results from GC-MS analysis

Of the 13 menthol-like substances selected for GC-MS analysis, seven were positively identified in one or more of the 18 investigated waterpipe products (Table 1). Menthol was frequently identified: 14 out of 18 products contained this flavoring. Menthol was present in relatively higher concentrations (0.22–7.01 mg/g) than the other investigated menthol-like components. Carvone was found in a broad range of relatively low (<0.07 mg/g) to high (2.34 mg/g) concentrations in the investigated products.

The products with ‘freeze’, ‘ice’ or ‘mint’ in their names/descriptions contained higher median concentrations of menthol (0.69 mg/g) than the products without ‘freeze’, ‘ice’ or ‘mint’ in their names (0.38 mg/g). For menthol-like ingredients, other than menthol, little difference was observed (median concentrations of 0.05 mg/g vs <0.07 mg/g).

Nicotine concentrations in waterpipe tobacco were between 0.91 and 1.54 mg/g. Steam stones S15 and S16 should contain nicotine according to information on the website and the sticker on the packaging material, but this was not detected by the GC-MS method.

## DISCUSSION

Our results show that nearly all investigated waterpipe products contained menthol-like compounds, irrespective of their notified flavor containing menthol or other descriptors referring to coolness (such as ‘ice’ or ‘freeze’). It is known from previous studies that flavorings such as menthol and some other menthol-like substances provide cooling effects by binding to the thermoreceptor TRPM8^10^. As such, they reduce the harshness of smoke and facilitate inhalation. However, an overview of products with menthol flavor in waterpipe tobacco was lacking, as was an overview of compounds imparting a menthol flavor or cooling effect. Furthermore, very few studies addressed the chemical identification of compounds similar to menthol, and these studies often lack confirmation with reference standards. Our work provides information on menthol and menthol-like substances that impart flavor and/or have cooling properties. We detected different cooling compounds in various waterpipe tobacco products. Since the TPD prohibits tobacco products with additives that facilitate inhalation, these compounds should not be present in waterpipe tobacco.

There is an increased supply of waterpipe tobacco for the Dutch market, based on the increased number, from 249 to 282, of EU-CEG registered waterpipe tobacco products between 2019 and 2020^19^. Our previous study looked at the most frequently used flavorings in waterpipe tobacco, such as vanillin, maltol and anethole^19^. Here, we focused specifically on menthol and compounds related to menthol by flavor or cooling properties in waterpipe tobacco. Twenty-two menthol-like substances were identified in the EU-CEG system. Frequently registered menthol-like substances were menthol, piperitone and cornmint oil. Menthol, carvone, and spearmint and cornmint oils, were registered as components in relatively high concentrations. Menthol and mint-oils are well-known, readily available, and relatively cheap flavorings to create a menthol/mint flavor, which likely explains their frequent use^7,26,27^. Earlier studies show that flavored waterpipe tobacco is preferred over non-flavored waterpipe tobacco, and that fruity flavors and menthol/mint flavors are the most preferred waterpipe tobacco flavors^18^.

We previously showed that menthol is added to all types of waterpipe products: tobacco, herbal molasses and steam stones^19^. In this study, it can be seen that the same is true for other menthol-like substances. Only 3/18 investigated products did not contain any of the investigated compounds. Products without menthol rarely contained other menthol-like substances. A possible explanation for this finding is the use of natural mint oils, which besides menthol contain varying levels of carvone, pulegone, isopulegol, piperitone, menthone, and menthyl acetate^28,29^. The only product in which a menthol-like substance was detected without the presence of menthol was product S8, which contained little carvone.

The highest concentrations were found for menthol compared to the other investigated substances. Carvone was also present in relatively high concentrations in some products. Other substances were present in relatively low concentrations. These findings are in accordance with the relatively high median concentrations of menthol and carvone in EU-CEG. Two European pharmacopoeia monographs describe the percentage content of mint oil (partly dementholized) and peppermint oil^26,27^. Menthol is usually present in 30–55%, and 6/7 of the identified menthol-like substances from this study (all besides piperitone) are present in varying quantities (0.2–35%), according to these monographs. Menthone is usually not found in a 1:2/1:3 ratio with menthol in our measurements, in contrast to what the mint oil composition would suggest, besides sample S5. It is possible that both pure menthol and mint oil are added to the same sample, which would alter the ratios of the detected components. Carvone is usually present in 1–2%, which is much lower than the ratio in which carvone and menthol are found in T7 and T8. This indicates that carvone was added separately or originates from another extract, such as caraway oil (50–65% carvone)^30^. A possible explanation of the presence of small amounts of menthol-like substances besides menthol is the use of mint oils^28^.

Nicotine concentrations in waterpipe tobacco in this study (0.91–1.54 mg/g) were similar to concentrations (0.65–1.8 mg/g) found by Erythropel et al.^31^.

Surprisingly, two steam stone products that should contain nicotine according to information on the website and the sticker on the packaging material lacked nicotine. This brand is sold with and without nicotine, and we expect that the vendor did not add any nicotine to these products in contrast to the indication on the label. The fact that both products lacked nicotine raises the question of whether the vendor does this more frequently, or that it was just forgotten for this specific order. We do not expect the added nicotine levels to be below our detection limit (0.1 μg/g), since this would be more than a thousand-fold lower than nicotine concentrations in waterpipe tobacco, which usually contains 0.5–2 mg/g (see Results section). The vendor website or product label did not contain information on the amount of nicotine in the steam stone sample. It remains unclear whether steam stones with added nicotine are available on the Dutch market.

Menthol, menthone, carvone and isopulegol are known TRPM8 thermoreceptor activators^7^. For menthyl acetate, pulegone and piperitone little is known about their cooling properties through TRPM8 receptor activation. However, menthyl acetate has been used for its cooling properties in flavored and perfumed products^7^. Also, pulegone reduces pain by binding to the TRPM8 receptor^8^. For piperitone, not much is known about cooling properties, even though piperitone has high structural similarities to other TRPM8 agonists.

Additional research, such as *in silico* or *in vitro* studies, would be needed to get a better insight in TRPM8 agonists. Also, sensory studies and/or questionnaires among users will provide a better insight into the perception (of flavoring or cooling effects) of the investigated menthol-like substances, to better understand the effect of adding these compounds to waterpipe and other products^32^.

Finally, it is essential to understand these substances’ possible side effects or harmfulness. For instance, it is known that pulegone is carcinogenic^33^. There are also indications that the fragrances mintlactone and menthofuran, which are also natural metabolites from pulegone, might also be toxic, but this requires further investigation^34,35^.

Menthol and various menthol-like compounds have cooling properties, which can reduce the harshness of the smoke and facilitate inhalation of tobacco smoke. Compounds that facilitate inhalation or nicotine uptake are already prohibited by the TPD (Articles 7.6 and 20.3) in tobacco products and e-cigarettes. Also, the US Food and Drug Administration (FDA) proposed to extend the definition of characterizing flavor with sensory experiences such as cooling sensations^36^. Banning product descriptors such as ‘cool’, ‘ice’ or ‘mint’ would reduce the attractiveness of tobacco and related products. In this study, we found that products, with and without product names or descriptions referring to the taste of cooling properties of menthol, contained menthol and related compounds. Therefore, products without menthol-related terms in their names or descriptions should still be screened for the presence of cooling compounds. Stricter regulation on menthol and similar compounds in all types of waterpipe products might reduce the addictiveness, attractiveness and harmfulness of waterpipe products and consequently discourage youth from smoking.

### Limitations

EU-CEG analysis is a valuable source of information on the chemical composition of additives in tobacco and nicotine products. However, non-tobacco based waterpipe products are often not registered in EU-CEG. Furthermore, EU-CEG data are manually entered by the manufacturer or importers, and might contain errors that can be incomplete or outdated. Therefore, waterpipe tobacco ingredients or median levels presented in this article might deviate from the products currently sold in The Netherlands.

Due to limited availability, only one herbal waterpipe product could be obtained and investigated. Therefore, based on our findings, providing a clear conclusion on this product class is difficult. However, menthol and some related compounds exist as different stereoisomers, (stereo) isomers in our GC-MS analytical method overlap and are not separated. This might be relevant for compounds of which only one stereoisomer provides a menthol-like flavor (such as R-carvone).

## CONCLUSIONS

Menthol or menthol-like compounds provide a cooling effect, thereby reducing irritation and facilitating inhalation during smoking. Of the categorized EU-CEG registered waterpipe tobacco products for the Dutch market, 39% contained menthol/mint in their product names or flavor descriptions. Most of the investigated products contain menthol or menthol-like compounds, regardless of their product names. Thus, regulators in jurisdictions where such compounds are forbidden should also screen waterpipe products without menthol in their product names.

## Data Availability

The data supporting this research are available from the authors on reasonable request.
