# Supplementary material for: Menthol and related compounds in waterpipe products
Source: Tob Prev Cessat. 2024 Feb 9;10:10.18332/tpc/177170. doi: 10.18332/tpc/177170 (PMC10854199; doi:10.18332/tpc/177170)
Supplement: Supplementary file 1 [file TPC-10-09-s1.pdf]

Supporting information

Menthol and related compounds in waterpipe products

Ingrid M.E. Bakker-'t Hart, Frank Bakker, Jeroen L.A. Pennings, Reinskje Talhout

Centre for Health Protection, National Institute for Public Health and the Environment, Bilthoven, Utrecht, The Netherlands

Table S1. EU-CEG registered menthol-like substances in waterpipe tobacco and their frequency of addition and median concentrations (2020, The Netherlands). Flavor and smoke taste descriptions were collected from the Leffingwell database [1]. Flavor descriptions are based on the individual flavorings. The smoke taste is the taste of a flavoring in smoke observed after combustion of tobacco with flavoring additive(s).Substances included forGC-MS analysis are marked in grey.

	Menthol-like substances	CAS no	Frequency of addition (%)	Median concentration (mg/g)	Flavor Description	Smoke taste	Included for GC-MS
**1**	L-menthol	2216-51-5	20.21	0.500	String trigeminal cooling sensation; slight mint note	Cooling	Yes
**2**	Piperitone	89-81-6	10.28	0.013	Fresh strong herbaceous, minty (D-piperitone: strong, fresh herbaceous, minty odor; harsh mint taste)	No description (D-piperitone: minty, herbaceous, spicy, adds body)	Yes
**3**	Cornmint oil	68917-18-0	8.51	2.532	Peppermint like odor & flavor	Historically, this material has been commonly used with synthetic menthol to provide a "natural" mint note; useful in non-menthol cigarettes at 10-20 ppm; quality varies with source	No
**4**	Para-mentha-8-thiol-3-one (or buchu mercaptan)	38462-22-5	4.96	0.013	"Catty" aroma; dull fruity black currant flavor*	Sulfuraceous, fruity	Yes
**5**	1-p-menthen-4-ol (or 4-carvomenthenol)	562-74-3	4.96	0.007	Sweet, green, citrus, cooling, musty; slightly peppery woody notes*	Green, earthy musty	Yes
**6**	L-carvone	6485-40-1	4.96	2.327	Spearmint odor & taste	Carvone: L-isomer; sweet, spearmint, herbaceous. D-isomer: green, weedy, herbaceous	Yes, but used standard is D-carvone CAS 2244-16-8
**7**	Peppermint oil	8006-90-4	4.96	1.452	Sweet, fresh (minty) aroma and cooling taste	Minty, adds body, weak herbaceous; useful as a modifier for synthetic menthol; useful in non-menthol cigarettes at 5-25 ppm,	No
**8**	R-(+)-Pulegone	89-82-7	4.61	0.011	Herbaceous, minty, resinous odor and taste	Herbaceous, minty	Yes
**9**	DL-isomenthone	491-07-6	3.90	0.072	Strong herbaceous-minty odor; herbaceous, minty, bitter taste	Not available	No
**10**	Spearmint oil	8008-79-5	3.55	1.886	Minty, herbaceous, weedy	Minty, herbaceous	No
**11**	DL-menthol	89-78-1	2.84	13.500	Cooling; less cooling than (-)-	No description	No

					menthol, with musty-minty notes		
**12**	Mint oil (mentha arvensis extract)	90063-97-1	2.48	1.800	Not found	Not found	No
**13**	N-ethyl-p-menthane-3-carboxamide (WS-3)	39711-79-0	1.77	0.468	Menthol like cooling sensation; flavor & salt enhancer	Cooling, like menthol	Yes
**14**	Spearmint absolute	84696-51-5	1.42	4.450	Strong, characteristic spearmint, slight-green-herbaceous	Minty, herbaceous, weedy, adds body	No
**15**	Mintlactone	13341-72-5	1.06	0.100	Sweet lactone-like note, a distinct coumarin and coconut note*	Coumarin-like; tends to fade on tobacco	Yes
**16**	Menthofuran	494-90-6	1.06	0.200	Sweet, musty-herbaceous, hay-like, minty odor; somewhat bitter taste	Bitter, harsh, minty, cooling	Yes
**17**	L-menthol ethylene glycol carbonate	156324-78-6	0.71	0.030	Provides trigeminal cooling similar to menthol	No description	No
**18**	Menthol 1- and 2-proypylene glycol carbonate	30304-82-6	0.71	0.034	Provides trigeminal cooling similar to menthol	No description	No
**19**	L-menthyl lactate	18915-25-7	0.71	0.034	Mild cooling sensation; sweet menthol taste	Very weak cooling sensation	Yes
**20**	Isopulegol	89-79-2	0.71	0.004	Cooling; minty, herbaceous; odorless & cool & fresh if highly purified	Bitter, herbaceous, resinous	Yes
**21**	Menthone	89-80-5	0.71	0.016	Cool, minty (minty-herbaceous (not green); dry woody notes	Not available	Yes: but used standard is L-menthone CAS 1407-97-3
**22**	L-menthyl acetate	2623-23-6	0.35	0.492	Sweet, fruity-sour, weak, minty cooling sensation	Sweet, floral, herbaceous, weak fruity; widely used in conjunction with menthone as a modifier for synthetic menthol in an attempt to impart a "natural menthol character	Yes

*thegoodscentscompany.com has the following odor descriptions for para-mentha-8-thiol-3-one (entry 4) " sulfury **minty** green fruity berry buchu fruity tropical peach" and entry 15 "powdery tobacco **spearmint**" and the following flavor description for entry 5: "cooling **menthol-like** woody weedy earthy herbal spicy citrus".

1. Leffingwell&Associates, *Flavor-Base 9 - Tobacco Version for Windows XP/Vista/7&8 2013*.

© 2024 Bakker-'t Hart I. M. E. et al.
